# Testicular Cancer Treatments and Sexuality: A Narrative Review

**DOI:** 10.3390/medicina60040586

**Published:** 2024-03-31

**Authors:** Massimiliano Raffo, Angelo Di Naro, Luigi Napolitano, Achille Aveta, Simone Cilio, Savio Domenico Pandolfo, Celeste Manfredi, Chiara Lonati, Nazareno Roberto Suardi

**Affiliations:** 1 Department of Urology, Spedali Civili Brescia, 25123 Brescia, Italy; chiara.lonati@libero.it (C.L.); suardi.nazareno@gmail.com (N.R.S.); 2 Division of Experimental Oncology, Unit of Urology, Urological Research Institute, IRCCS Ospedale San Raffaele, 20132 Milan, Italy; 3 Faculty of Medicine, Vita-Salute San Raffaele University, 20132 Milan, Italy; 4 Department of Neurosciences, Science of Reproduction and Odontostomatology, University of Naples Federico II, 80138 Naples, Italy; nluigi89@libero.it (L.N.); achille-aveta@hotmail.it (A.A.); simocilio.av@gmail.com (S.C.); pandolfosavio@gmail.com (S.D.P.); 5 Urology Unit, Department of Woman, Child and General and Specialized Surgery, Luigi Vanvitelli University, 80138 Naples, Italy; dott.celestemanfredi@gmail.com

**Keywords:** testicular cancer, seminoma, non-seminoma, molecular markers, lymph node metastasis, diagnosis, systemic treatment

## Abstract

The incidence of testicular cancer (TC) has been rapidly increasing over the past years. Diagnosis and early treatment have shown good oncological control, guaranteeing the patient different treatment approaches according to histology and tumor stage. Currently, physicians usually prioritize oncological outcomes over sexual outcomes and quality of life, considering as a first aim the overall survival of the patients; however, differently from other neoplasms, quality of life is still strongly affected among TC patients, and sexual outcomes are frequently compromised after each TC treatment. Several studies have suggested that each treatment approach may be associated with sexual dysfunctions, including erectile dysfunction, ejaculatory disorders, fertility issues, and hormonal changes. Since testicular cancer patients are more frequently young men, the subject of this work is substantial and should be analyzed in detail to help specialists in the management of this disease. The aim of the current narrative review is to generally describe every treatment for TC, including surgery, chemotherapy, radiotherapy, and retroperitoneal lymph node dissection, and to establish which sexual dysfunction may be specifically associated with each therapy.

## 1. Introduction

Cancer is the main cause of death worldwide, alongside cardiovascular diseases [1]. Roughly 5% of the population has received a diagnosis of cancer [2]. However, the life expectancy of about 60% of young adult and juvenile cancer survivors can be compared to that of the general population, thanks to modern treatment [2]. Men of reproductive age are mainly affected by lymphomas and testicular cancer (TC), and the 5-year survival rates of these patients are above 80–90% [3]. Although TC can be considered a rare malignancy among men of all ages [4], accounting for 1% to 2%, considering men between 20 and 40 years of age [4,5,6], TC is the most common neoplasm [7,8]. Over the last 50 years, the incidence of the disease has massively increased in North America and in Northern Europe [9,10]. Twenty-five to thirty-five percent of the patients show distant metastasis at first presentation [4]; thanks to the combination of surgery, chemotherapy, and radiotherapy, excellent long-term survival rates can be achieved [11,12]. The diagnosis of TC can be achieved thanks to the self-palpation and ultrasonography of the testes, which typically shows a hypoechoic intratesticular lesion or a heterogeneous irregular mass [13,14]. Serum alpha-fetoprotein (AFP), beta subunit of human chorionic gonadotropin (β-hCG), and LDH should be executed before and after orchidectomy as they guide the diagnosis of TC and may be indicative of germ cell tumor histology. Up to 90% of non-seminomatous germ cell tumors have elevated AFP or β-hCG at diagnosis, with 39% having an increased level of both. Pure seminomas may also have modestly elevated β-hCG levels at diagnosis in up to 30% of cases. Significant elevation of AFP in patients with seminomas should raise concerns about a non-seminoma component. Modest stable elevations may be considered a normal variant. Tumor markers have limitations due to their low sensitivity as normal levels do not exclude the presence of disease.

Despite the high survival rate and the high rate of response to treatments [15], many TC patients experience a broad set of undesired social, psychological, and sexual side effects due to the treatments [16,17,18,19,20]. In particular, as part of the male reproductive system, the testes play a functional role in spermatogenesis and in the formation of testosterone by assisting in androgen production [21,22,23], and so, this can be impaired. The current literature does not comprehend nomograms or specific assessment measures that help physicians foresee sexual dysfunctions after testicular cancer treatments. So, before starting any therapeutic option, medical doctors must provide a complete clinical evaluation, including a score for comorbidities, such as the Charlson Comorbidities Index (CCI) [24], and questionnaires such as the International Index of Erectile Function (IIEF) [25]. In addition, every TC patient should be offered semen preservation as the most cost-effective strategy for fertility preservation. This should be offered before any type of treatment when feasible, maximizing the chances of fertilization and avoiding the risk of a non-functioning remaining testicle. If not arranged before orchidectomy, it should be undertaken prior to chemotherapy or RT [26,27,28,29,30].

The purpose of this review is to synthesize the current literature on the topic of sexual dysfunction after different treatments for TC. In particular, we want to analyze each treatment modality and its side effects on the sexuality of men who survived TC. The further aim of this narrative review is to explore which treatment for TC may affect sexuality and quality of life the least.

## 2. Search Strategies and Selection Criteria

A narrative review was conducted through an extensive literature search using the PubMed, EMBASE, and Google Scholar platforms. Medical Subject Heading (MeSH) terms and keywords such as “testicular cancer”, “sexual dysfunction”, “infertility”, “erectile dysfunction”, “ejaculation”, “surgery”, “radiotherapy”, “chemotherapy”, and “retroperitoneal lymph node dissection” were used. No date limits were imposed.

Titles and abstracts of the manuscripts were used to screen for initial study inclusion. Full-text review was performed when the abstract was not sufficient to determine study inclusion. Review articles, commentaries, editorials, and articles that did not undergo a peer review were excluded. Non-human studies were excluded from our research. Reference lists of included studies were hand-searched for completeness. For studies not available online, the authors were contacted to gain access to publications and data. The search and data collection were conducted independently by two authors (M.R. and A.D.N), and controversies were discussed with senior coauthors.

## 3. Sexual Dysfunction after Surgery

Patients suspected of having TC should undergo an orchiectomy with inguinal access to remove the testicle that contains the tumor and the spermatic cord at the level of the inguinal ring [31]. Alternatively, if the testicle can be spared, surgeons usually prefer to perform a testis-sparing surgery [32]. Since TC is associated with very rapid growth, surgery should be performed within one or two weeks from the diagnosis [33]. In addition, the trans-scrotal approach or a biopsy of the neoplasm must be avoided to not alter the lymphatic drainage of the testis and thus increase the risk of local recurrence and pelvic or inguinal lymph node metastasis. For what concerns partial orchiectomy (or testis-sparing surgery), it finds indication in organ-confined tumors of less than 2 cm in patients with synchronous bilateral tumors or tumors in a solitary testis with sufficient testicular androgen production [34,35,36]. Partial orchiectomy can also be considered for suspected benign tumors or indeterminate lesions with normal testicular marker values. Testis-sparing surgery is usually not possible for tumors bigger than 2 cm because a radical excision frequently leaves insufficient residual testicular parenchyma. Tumor markers should be repeated following surgery in order to consider re-staging and prognostic information. If elevated preoperatively, it may take several weeks to assess normalization as the serum half-lives of AFP and β-hCG are five to seven days and one to three days, respectively. If molecular marker levels remain elevated or increase, metastatic disease is likely. Molecular marker normalization after orchidectomy, however, does not exclude the possibility of metastatic disease.

In addition to staging, molecular marker levels are used to define risk stratification and prognosis. They are also used to monitor treatment response and detect disease relapse. With follow-up, the precise frequency of testing is not well defined.

For what concerns sexual disturbances, the act of surgery alone may lead to many side effects in patients treated for TC. In fact, orchiectomy may produce a decrease in self-esteem and a change in body aspect; in the paper of Incrocci et al., of 166 patients treated with surgery, 52% experienced a change in their body [37]. In addition, Schover et al. demonstrated that TC patients may experience pain, depression, physical debilitation, and anxiety, all of which may harm sexual drive [38]. In the narrative review conducted by Barros, it was shown that testicular surgery for cancer could harm patients in terms of attractiveness [39]; in fact, the authors stated that the disturbance in body image and sense of attractiveness were significantly modified after surgery [40]. Nezu et al. confirmed this theory, demonstrating that more than 30% of patients undergoing orchiectomy felt less attractive after surgery [41]. Regarding the theme of fertility, Elenkov et al. assessed that 10% of patients who underwent orchiectomy for TC reported a condition of azoospermia in semen analysis [42].

Relevantly, Palotti et al. [2] evaluated the possible effect of TC and orchiectomy on sexual function. They administered the IIEF-5 to TC patients at the post-orchiectomy baseline before chemotherapy and found that 37.7% of patients had erectile dysfunction. According to the authors, the sexual dysfunction in these patients might be associated with psychological burden. In fact, sexual dysfunction in TC is not clearly related to disease or treatment factors and may instead arise from psychological vulnerability [43].

## 4. Sexual Dysfunction after Chemotherapy

Primary systemic treatment with chemotherapy is the treatment of choice for patients at stage I TC, particularly for non-seminoma germ cell tumors. The difference between systemic treatment with chemotherapy and adjuvant chemotherapy is the fact that the latter is given to patients at stage II after retroperitoneal lymph node dissection (RPLND). The primary aim of a systemic treatment is to minimize the risk of relapse and to allow men to avoid RPLND and induction chemotherapy (for those who experience relapse on surveillance). The rationale for primary systemic treatment is based on the efficacy of two cycles of chemotherapy in eradicating micro-metastatic disease when given as adjuvant therapy after RPLND and the 20% to 25% need for chemotherapy despite RPLND (either as adjuvant or for treatment of relapse) [44,45,46]. Of course, primary chemotherapy is not free from disadvantages. In fact, it does not attack retroperitoneal teratomas, and thus, it exposes patients to chemoresistance and relapses; in addition, these patients may suffer from cardiovascular disease and secondary malignant neoplasms [47], although relapses are uncommon with primary chemotherapy. The three chemotherapeutic agents used for this kind of neoplasm are Bleomycin, Etoposide, and Cisplatin (BEP) [48,49,50,51,52,53]. Many European institutions prefer one cycle of BEP compared to RPLND because RPLND is primarily used as a staging procedure and performed without curative intent [54,55].

As a therapeutic strategy for TC, systemic treatment has an important impact on the sexuality of patients. Considering infertility, chemotherapeutic agents harm the rate of fecundation more than radiotherapy [56,57,58,59,60,61]. Averette et al. showed that combination chemotherapy in TC has substantial effects on gonadal function, giving almost all patients an azoospermic condition, and a high degree of recovery of spermatogenesis occurs sometimes after 2–3 years from the initiation of the therapy [62]. On the same line, Uçar MA et al. reported that following chemotherapy, sperm counts in almost 80% of patients return to their preoperative levels within 2–5 years, and after 3 years, most of them have reached fatherhood [63]. Other studies underline that the effect of chemotherapy has a different impact on spermatogenesis depending on the type of the drug and on the cumulative dose used; high doses of BEP may affect spermatogenesis and thus fertility [21,22]. Bujan et al., by analyzing 129 patients, reported that at least two cycles of BEP had detrimental effects; in fact, patients experienced a decreased semen volume reflecting a transient dysfunction of genital glands [64]. This effect may be correlated to some changes in hormone production. Ondrusova et al. analyzed hormonal changes in 313 patients who received orchiectomy alone, orchiectomy plus radiotherapy, and orchiectomy plus chemotherapy for testicular cancer [65]. They reported that luteinizing hormone levels were truly decreased in testicular cancer patients who received chemotherapy after surgery [65].

Always in the context of sexual dysfunctions, Bokemeyer et al. showed that systemic treatment for TC was related to the absence of ejaculation due to damage to hormone production and the vascular and nervous systems [66]. This result is in line with other studies reporting alteration of ejaculation [10,67]; van Basten analyzed 43 patients treated with a systemic treatment and showed that 17.4% exhibited a decreased semen amount and 18.7% exhibited a complete absence of antegrade ejaculation [10]. A decreased erection potential was also found in patients treated with chemotherapy [36,43,67,68,69]. In 2018, Bandak et al. demonstrated that the risk of erectile dysfunction in 2260 long-term survivors of testicular cancer compared to surveillance was significantly higher [68]. Sexual desire and male disappointment have been importantly considered as side effects of this kind of treatment option [36,67,68]. Orgasmic dysfunction may depend on several factors such as radiotherapy or chemotherapy; in fact, they provoke neuropathy, lower levels of testosterone related to psychological issues, and the possible use of selective serotonin inhibitors [47]. These treatments do not harm patients in every case; in fact, in the previously cited study, Bandak et al. demonstrated that patients treated with BEP for testicular germ cell tumors have a preserved orgasmic phase [68].

## 5. Sexual Dysfunction after Radiotherapy

To prevent the relapse of a germ cell tumor, adjuvant radiotherapy to partial orchiectomy to the residual testis can be an important strategy in the treatment of TC, even if it is not recommended in the current guidelines [49]. The German Testicular Cancer Study Group reported no cases of local recurrence over a median follow-up of 91 months in 46 patients with small, organ-confined tumors who underwent testis-sparing surgery and received adjuvant radiotherapy for ITGCN [70]. Until recently, the gold standard of the treatment for seminoma stage I for the past 4 decades had been primary radiotherapy of the retroperitoneum and ipsilateral pelvis. Overall, long-term cancer-specific survival approaches 100%, while progression-free survival rates range from 95% to 97% [34,71,72]. The recurrence rate after radiotherapy in patients treated for TC is less than 1%, obviating the need for routine surveillance abdominal–pelvic CT imaging. Inguinal metastases are uncommon among patients without prior inguinal or scrotal surgery. Patients with isolated inguinal relapse may be treated with radiotherapy or surgical resection. The surveillance of patients after radiation therapy consists of regular clinical assessment, chest X-rays, and serum tumor markers. Many patients experience some acute side effects with adjuvant radiotherapy, which typically include transient nausea, vomiting, and diarrhea. Acute grade II-IV hematologic toxicity occurs in 5% to 15% of patients [72,73]. Moderate and severe late gastrointestinal toxicity (usually chronic dyspepsia or peptic ulcer disease) is reported in 5% and less than 2% of patients, respectively.

Regarding sexual dysfunction related to radiation therapy, scientists tried to analyze the potential side effects in the field of TC. Comparing radiation to other treatments (such as chemotherapy and retroperitoneal lymph node dissection), Rieker et al. demonstrated that patients treated with ionizing radiation experienced fewer sexual dysfunctions (considering symptoms like erectile issues and psychosocial problems) [57]. On the same line, Shover et al. reported that seminoma patients treated with radiation therapy were less likely to experience a severe reduction in orgasmic pleasure: this may be explained by the fact that radiotherapy interferes less with the male orgasmic phase compared to the other treatment options [56]. In addition, Arai et al. reported that the radiotherapy group of patients showed an increased rate of side effects in the field of erectile function and premature ejaculation [74]. The theme of erectile dysfunction can be considered an important issue considering radiotherapy for TC. The reasons behind this phenomenon have not been fully elucidated; potentially, radiation may harm Leydig cell function, as well as testosterone production, leading to erectile dysfunction [10,43]. In 2019, La Vignera et al. showed in their systematic review that patients irradiated for TC experienced a reduction in testosterone levels, which resulted in erectile dysfunction [75,76,77,78]. The decrease in testosterone levels after radiotherapy was also analyzed by Ondrusova et al. [65]; interestingly, they found that ionizing radiation did not affect LH levels [65]. On the other hand, Pallotti et al. demonstrated in their retrospective study that erection improves one year after the treatment, becoming almost normal after two years in these patients [2]. Many investigators also demonstrated that a reduced amount of semen volume was observed in the radiotherapy group compared to surgery and chemotherapy ones [79]. However, radiotherapy patients had the lowest rate of infertility distress compared to the other groups [57]; in fact, in a retrospective analysis of 223 TC patients, worse semen parameters in terms of sperm count were found in the ones treated with systemic treatment [57].

## 6. Sexual Dysfunction after RPLND

The retroperitoneum is the initial site of lymph node metastasis in 70% to 80% of patients with TC [80]; the most common dissemination pathway is via lymphatic channels from the primary tumor to the retroperitoneal lymph nodes and subsequently to distant sites. The only exception is for choriocarcinoma, which has a propensity for hematogenous dissemination. Lymph node metastasis for TC has been understood by studies conducted from the RPLND allowing scientists to identify the most likely sites of metastatic spread [81]. Inter-aortocaval lymph node metastasis inferior to the renal vessels is the primary drainage site for right testicular tumors, followed by the paracaval and para-aortic nodes. The primary zone for left testicular tumors is the para-aortic lymph nodes, followed by the inter-aortocaval nodes [44]. All patients with TC should undergo staging imaging of the abdomen and pelvis to explore and exclude lymph node metastasis [82]. CT after administration of contrast agents is the most effective, non-invasive means of staging the retroperitoneum and pelvis. CT also provides a detailed anatomic assessment of the retroperitoneum to identify anatomic anomalies that may complicate subsequent RPLND [83,84]. Magnetic resonance imaging (MRI) is an alternative to CT. Thus, the rationale for RPLND in TC patients is related to the following: (1) the retroperitoneum is the most common site of metastasis; (2) 15% to 25% incidence of retroperitoneal teratoma (which is chemo-resistant) in those with occult metastasis is found in the retroperitoneum [85,86,87]; (3) RPLND may lead to low risk of abdominopelvic recurrence [84,88]; (4) avoidance of chemotherapy in more than 75% or more of patients if adjuvant chemotherapy is restricted to those with extensive retroperitoneal malignancy (pN2-3) [89,90,91,92]. The disadvantages of RPLND are that all patients undergo major abdominal surgery, it requires the availability of experienced surgeons and thus may not be deliverable to all patients, and it is associated with the highest rate of double therapy [93,94]. Roughly, 25% of non-seminoma patients undergo RPLND during the therapeutic pathway [95,96,97,98].

From the literature, we can surely report that the quality of sexual life is quite reduced in TC survivors; in particular, 40% of the patients are dissatisfied with their sexual life [38,99]. Considering sexual function, the most important complication of RPLND is the loss of antegrade ejaculation. RPLND has the potential to damage the postganglionic sympathetic fibers from the lumbar splanchnic nerves (L2–L4), leading to an antegrade function [5,41,100,101] or anejaculation [102]. During the 1990s, the lymph nodal dissection template was modified with nerve-sparing techniques in order to prevent this sexual dysfunction [41,103,104]. This technique, by preserving nerve bundles, can help in maintaining a patient’s sexuality with little changes. In fact, Cogo Badan et al., in their case report, showed that after RPLND with a nerve-sparing technique, ejaculation was preserved [105]. Another study confirmed this theory; in fact, by analyzing 19 patients treated with nerve-sparing RPLND, McClintock et al. assessed a full recovery of anterograde ejaculation [106]. In 1994 and 2008, Miki et al. also reported that, of 92 TC survivors, 83.1% had normal antegrade ejaculation with a median period of 3 months after nerve-sparing RPLND [107].

Although the advent of nerve-sparing techniques and modified templates reduced the sexual side effects of RPLND, the procedure itself continued to have significant morbidity, and it cannot be performed in every TC patient [108]. For example, comparing systemic treatment and RPLND, ejaculatory issues were more represented in the surgery group [109]; this fact can be related to non-nerve-sparing techniques. These effects were reported by Koyama et al. in their cross-sectional study where they analyzed 567 patients treated for TC [109]. In line with that, ejaculatory function after RPLND was better with nerve-sparing techniques than without. Many other studies confirmed that RPLND has an important effect on ejaculation disorders [21,22,110,111]. Also, response rates for ejaculatory function were much higher in the nerve-sparing group compared to the non-nerve-sparing group. In addition, TC survivors with nerve-sparing RPLND showed higher sexual activity than those without [109].

Back to sexual dysfunctions, 20–30% of patients reported reduced intensity of orgasm according to loss of libido conditioning the inability to ejaculate after RPLND [112]. Mistretta et al. reported that of 32 patients treated with bilateral RPLND, 12% experienced dry ejaculation [113].

In addition, Matos et al. stated that non-nerve-sparing techniques showed important issues of fertility [114,115,116]. Another important issue regarding surgery is erectile dysfunction; contrary to what can be hypothesized, Dimitropoulos et al. showed that in 53 patients, no difference in erectile functioning before and after operation was found [5].

## 7. Conclusions

In conclusion, the wide variety of TC treatments guarantee a complete eradication of the tumor, resulting in a very high rate of survival for young patients. However, the risk of adverse events concerning sexuality is quite high and depends on the type of treatment. On one hand, orchiectomy and RPLND are mostly associated with psychological issues such as a decrease in self-esteem and erectile dysfunction. On the other hand, systemic therapies and radiotherapy involve an increased risk of hormone disbalance leading to fertility issues and ejaculation disorders.

Therefore, patients need to be counseled about the likely changes in sexual function before each type of treatment for testicular cancer. Firstly, urologists, with the cooperation of oncologists, should systematically inform, educate, and comfort these patients during the treatment. Multidisciplinary medical teams, including sexual medicine physicians and psycho-oncologists, play a fundamental role in this scenario and need to be proactive by offering psychological support to mitigate the impact on male sexuality. However, more studies are needed to clarify the impact testicular cancer and its treatments may have on the sexual function of men, and clinicians need better training about the best way to approach these issues.

## 8. Future Directions

Future research in this field is warranted to explore the underlying the exact mechanisms of sexual dysfunctions compared to each treatment in the field of TC. In addition, in the revision of guidelines, a complete nomogram has to be created to assess the risk of developing any sexual disorder after being treated for this kind of neoplasm.

## Data Availability

All data are derived from other studies. No original data are available from the corresponding author.
